# Treatment of Chronic Pain Due to Slipping Rib Syndrome Using Ultrasound-Guided Intercostal Cryoneurolysis: A Case Report

**DOI:** 10.1155/cria/8800687

**Published:** 2025-03-11

**Authors:** Igor Filipovski, Freja Vesterdahl, Brian P. Curran, Rodney A. Gabriel

**Affiliations:** ^1^Copenhagen Cryo Center, Copenhagen, Denmark; ^2^University of Copenhagen, Copenhagen, Denmark; ^3^Division of Regional Anesthesia, Department of Anesthesiology, University of California, La Jolla, San Diego, California, USA

## Abstract

Slipping rib syndrome (SRS) is an underdiagnosed condition, in which some ribs are not connected to the sternum, which may cause increased laxity of the interchondral ligament. This may result in pain in the lower chest and upper abdomen area. Treatment typically includes conservative measures, steroid injections, and surgery. Ultrasound-guided intercostal cryoneurolysis is a minimally-invasive procedure that may provide long-term analgesia in patients with SRS. This case report describes the procedure for a patient treated with right-sided ultrasound-guided cryoneurolysis of the intercostal nerves at the levels T7, T8, and T9.

## 1. Introduction

Slipping rib syndrome (SRS) is an underdiagnosed condition that causes pain in the lower chest and upper abdomen [1]. SRS is caused by either a congenital deformity of the anterior “false” ribs (typically 8th–10th) or disruption of fibrous articulation leading to a hypermobility that allows the 8th–10th rib to slip under the rib above and put pressure on the intercostal nerve. The impingement of the intercostal nerve can cause local pain in the area surrounding the curvature and/or the upper abdomen [2]. The initial treatment for SRS involves conservative management and consists of nonsteroidal anti-inflammatory drugs, rest, ice, physical therapy, massage, or chiropractic manipulation. If the conservative treatment is unsuccessful, minimally-invasive treatment with a combination of steroids and local anesthetics may be considered. If none of the above treatments prove successful, the patient may undergo surgical intervention with resection of the subperiosteum and costal cartilage [2]. Cryoneurolysis may be an alternative, less invasive option for analgesia in patients with SRS whose pain is refractory to conservative measures. Several studies and case reports have described the use of ultrasound-guided intercostal cryoneurolysis to treat chronic truncal pain [3]. The patient described in this case report provided written consent for performance of the procedures and consent for publication, and an Institutional Review Board approval was not required. Health Insurance Portability and Accountability Act authorization was obtained.

## 2. Case Report

A 34-year-old man presented with bilateral pain on the anterior chest wall. The patient had undergone a computed tomography scan with no pathological findings. The pain started on the left side of the anterior wall and spread to the right side 1 year later. The patient experienced pain under both curvatures when bending forward and a feeling of the inferior ribs being trapped. On the visual analog score (VAS), the patient reported a pain intensity of 40 mm, which worsened with increased physical activity. The patient was then referred to the pain clinic by a thoracic surgeon after being diagnosed with bilateral SRS.

Upon his first visit to the clinic, the patient received an ultrasound-guided diagnostic block. The left intercostal nerve at the T11 level was located in the midaxillary line using ultrasound. Ropivacaine 5 mg/mL (1 mL) was injected surrounding the nerve. The same procedure was repeated with the left intercostal nerve at T12. There was an instant effect, and the pain relief lasted 40 min. The pain on the left side disappeared, but the patient experienced pain in an area superior to the dermatome of T11. There was no effect on the pain on the right side.

A few weeks later, the diagnostic block was repeated on the intercostal nerves at the T10, T11, and T12 levels bilaterally using mepivacaine 10 mg/mL (1 mL per level). The patient had a very good analgesic response for a few hours and reported that all the pain affected area was covered by this block.

Subsequently, the patient was involved in an accident, and the treatment was paused for 6 months. When the treatment was resumed, the patient then described pain consistent with the dermatomes of the intercostal nerves at the T8, T9, and T10 levels, and the pain was now worse on the right side. The patient reported an increase in pain intensity of 60 mm on VAS. The diagnostic block was repeated on the right intercostal nerves at the T8, T9, and T10 levels using mepivacaine 10 mg/mL (1 mL per level). Patient reported 100% analgesia thereafter.

**Figure 1:**
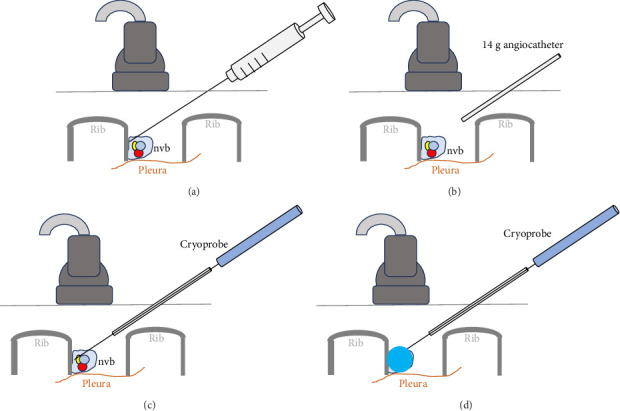
(a) Intercostal nerve block; (b) insertion of 14-g angiocatheter to serve as an introducer; (c) insertion of the 16-g cryoprobe through the introducer and then placed adjacent to the intercostal nerve; and (d) ice ball formation at the neurovascular bundle created by the cryoprobe. Abbreviation: nvb = neurovascular bundle.

**Figure 2:**
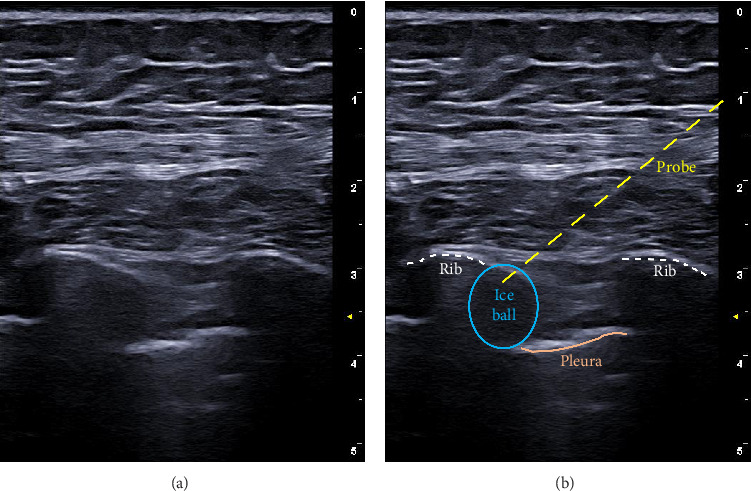
Ultrasound image of the intercostal cryoneurolysis procedure for this patient: (a) unlabeled ultrasound image and (b) labeled ultrasound image. The dotted white lines refer to the ribs, the orange line is the pleura, the dotted yellow line is the cryoprobe, and the blue circle is the ice ball generated by the cryoneurolysis. For this procedure, the patient was placed in the lateral decubitus positon, and the ribs were identified at the midaxillary line.

At the next visit to the clinic, the patient reported that the pain had moved more superior to the dermatome of T7 and was worse on the right versus left side. The patient was placed in the left lateral decubitus position, and the procedure was performed in the midaxillary line. Prior to cryoneurolysis treatment, the right-sided T7–T9 intercostal spaces were injected with mepivacaine 10 mg/mL (1 mL per level) (Figure 1(a)). The patient reported complete analgesic coverage on the right side. Thus, a 14-g angiocatheter needle was then inserted proximal to the T9 intercostal nerve (Figure 1(b)). The needle was disengaged, and the cryoprobe was inserted through the angiocatheter, which served as an introducer (Figure 1(c)). The probe (1.3 triangular shaped needle) was placed deep near the intercostal space, above the pleura, and in contact with the right-sided T7 rib. Here, two 3-min freezing cycles (with 1-min thaw period in between) were performed (Metrum Cryoflex, Warsaw, Poland) (Figure 1(d)). This was then repeated for the intercostal nerves at the T8 and T9 intercostal spaces. Immediately after the procedure, the patient reported a 100% analgesic effect. No cryoneurolysis was performed on the left side (Figure 2).

At the follow-up consultation 3 weeks and 4 months after, the patient reported a pain intensity of 0 mm on the VAS on the right side. His functional level was overall improved, and he was able to perform more activities in his everyday life. The patient's quality of life was improved; however, he still experienced pain in the left side of the anterior chest wall. Immediately following the procedure and at follow-up, the patient did not report any side effects from the treatment, including muscle laxity of the rectus abdominis or paresthesias. At all follow-ups, tests with ice and pinprick to skin demonstrated no changes in sensory function. Although we did not measure formal lung function and capacity postprocedure, the patient reported less discomfort with deep inhalation.

## 3. Discussion

The treatment options for SRS are limited, and the analgesia provided by conservative treatment often only has short-term effects. This case report examines the viability of ultrasound-guided cryoneurolysis in the treatment of pain related to SRS. The patient had a beneficial response from the treatment in regards to analgesia, physical activity, and quality of life. Diagnosing SRS may be challenging and often misdiagnosed. Accurate diagnosis includes clinical and imaging tests. Key symptoms include clicking, popping, or snapping sensation of the ribs. On physical exam, a hooking maneuver test can be performed to suggest SRS. Finally, imaging exams are not always accurate but can be used to rule out other conditions—chest X-ray may not show any abnormalities, dynamic ultrasound may potentially visualize excessive rib movement, and MRI/CT scans may detect costal cartilage degeneration [4].

Cryoneurolysis is a technique of peripheral neurolysis using extremely cold temperatures (no colder than −100ºC) [5]. This intervention may be applied intraoperatively (e.g., via surgical approach during intrathoracic surgery) [6] or percutaneously via ultrasound-guidance or landmark approaches with or without peripheral nerve stimulation. The tip of the cryoprobe is then positioned adjacent to a target nerve. At initiation of treatment, a gas, such as nitrous oxide, carbon dioxide, or argon, travels down the center of the probe, where it escapes through a small opening, which causes a Joule–Thomson effect—a thermodynamic principle that describes how a gas temperature changes when it expands [7]. The temperature cools as the gas expands, which subsequently creates the ice ball. Notably, these resulting cold temperature (if no colder than −100ºC) destroys only the myelin sheath and not the endoneurium. As the nerve is not completely destroyed, formation of a neuroma is prevented, while also reducing nerve signaling of pain due to the reversible destruction of the myelin sheath [8]. Full regeneration of the myelin sheath occurs over days to months at approximately 1-2 mm per day. Based on animal studies, cryoneurolysis provides prolonged analgesia with the eventual regeneration of a nerve with return to baseline motor and sensory function [9]. This provides a window of painless rehabilitation and then the return of full nerve function [10].

For this patient, symptom reduction was achieved but due to the singularity of the case report, further research needs to be conducted to examine proof of generalizability and long-term efficacy of ultrasound-guided cryoneurolysis for SRS. If demonstrated to be efficacious, this would provide a less invasive (relative to surgery) analgesic option for this population. Cryoneurolysis of the intercostal nerve may be considered as an analgesic modality for patients with SRS. Pulse radiofrequency ablation is a similar modality that may target the nerve but has a differing mechanism, in which short bursts of electrical energy and high frequency alter pain signal transmission without completely ablating the nerve. Both this and cryoneurolysis approaches are minimally invasive and may potentially be used for intervention of SRS, although more studies are needed to compare efficacy and outcome between both interventions. Further research needs to be conducted to explore the long-term effects and the heterogeneity in the treatment of SRS.

## Data Availability

Deidentified data may be made available upon reasonable request and with appropriate data use agreements in place.
